# Primary or Interval Debulking Surgery for Advanced Endometrial Cancer with Carcinosis: A Systematic Review and Individual Patient Data Meta-Analysis of Survival Outcomes

**DOI:** 10.3390/cancers17061026

**Published:** 2025-03-19

**Authors:** Giulia Mantovani, Camelia Alexandra Coada, Stella Di Costanzo, Francesco Mezzapesa, Lucia Genovesi, Giorgio Bogani, Francesco Raspagliesi, Alessio Giuseppe Morganti, Pierandrea De Iaco, Anna Myriam Perrone

**Affiliations:** 1 Division of Oncologic Gynecology, IRCCS Azienda Ospedaliero-Universitaria di Bologna, Via Massarenti 9, 40138 Bologna, Italy; giulia.mantovani18@unibo.it (G.M.); francesco.mezzapesa@studio.unibo.it (F.M.); lucia.genovesi@studio.unibo.it (L.G.); pierandrea.deiaco@unibo.it (P.D.I.); myriam.perrone@unibo.it (A.M.P.); 2 Department of Morpho-Functional Sciences, University of Medicine and Pharmacy “Iuliu Hațieganu”, 400347 Cluj-Napoca, Romania; camelia.coada@unibo.it; 3 Department of Medical and Surgical Sciences (DIMEC), University of Bologna, 40138 Bologna, Italy; alessio.morganti2@unibo.it; 4 Gynecologic Oncology Unit, Fondazione IRCCS Istituto Nazionale dei Tumori, 20133 Milan, Italy; giorgio.bogani@istitutotumori.mi.it (G.B.); francesco.raspagliesi@istitutotumori.mi.it (F.R.); 5 Radiation Oncology, IRCCS Azienda Ospedaliero-Universitaria di Bologna, 40138 Bologna, Italy

**Keywords:** endometrial cancer, peritoneal carcinomatosis, primary debulking surgery, interval debulking surgery, platinum-based neoadjuvant chemotherapy, platinum-based adjuvant chemotherapy

## Abstract

Objective. To compare the survival outcomes of primary debulking surgery and platinum-based adjuvant chemotherapy versus interval debulking surgery after platinum-based neoadjuvant chemotherapy in patients with stage IVb endometrial cancer and peritoneal carcinosis. Methods. The online search included the following data sources: PubMed, Scopus, WOS, and the Cochrane Library from 1990 to 2024 (PROSPERO registration code: CRD42023438602). A total of 3230 studies were identified, with the inclusion of 16. Individual patient data on survival outcomes, disease distribution, and residual tumors, as well as details of neoadjuvant chemotherapy and adjuvant treatment, were extracted. Results. A total of 285 patients were included: 197 (69%) underwent primary debulking surgery and 88 (31%) underwent interval debulking surgery. The pooled analysis revealed a median progression-free survival in the primary debulking surgery group of 18.0 months compared to 12.0 months in the interval debulking surgery group (*p* = 0.028; log-rank test), and a median overall survival of 30.92 months versus 28.73 months (*p* = 0.400; log-rank test). Among the 134 patients with available information on the residual tumor after primary debulking surgery or interval debulking surgery, 110 (82%) had no macroscopic residual tumor (residual tumor = 0). The median progression-free survival was 18.9 months in the residual tumor = 0 group compared to 6.19 months in the residual tumor > 0 group (*p* < 0.001; log-rank test); the median overall survival was 40.6 months versus 21 months (*p* = 0.028; log-rank test). Conclusions. These results indicate that primary debulking surgery should be considered the preferred treatment approach for advanced endometrial cancer with carcinosis, especially in carefully selected patients where complete cytoreduction is achievable. Further prospective studies are warranted to confirm these results and to establish standardized criteria for patient selection, incorporating molecular-integrated risk profiles for endometrial cancer.

## Simple Summary

The role of surgery in stage IV endometrial cancer with peritoneal carcinosis remains unclear. This systematic review and individual patient data meta-analysis compares two treatment approaches: primary debulking surgery followed by platinum-based chemotherapy, versus interval debulking surgery after neoadjuvant platinum-based chemotherapy. The analysis included 285 patients and found that primary debulking surgery resulted in longer progression-free survival (18 months vs. 12 months) but similar overall survival (around 30 months for both groups). The presence of no residual tumor after surgery was associated with better survival outcomes. This study suggests that primary debulking surgery should be considered the preferred option for advanced endometrial cancer with peritoneal metastasis when complete tumor removal is possible. Further studies are needed to refine treatment selection, potentially using molecular risk profiles.

## 1. Introduction

Endometrial cancer is the most common malignancy of the female genital tract in high-income countries, with an annual incidence of over 420,000 cases worldwide and an age-standardized rate of 15.5 per 100,000 women in Europe [1]. The disease is usually diagnosed when confined to the uterus; however, a proportion of patients have regional lymph node involvement (20%) or distant metastases (10%) at the time of diagnosis [2]. Stage IV endometrial cancer includes a heterogeneous patient population who may present with disease confined to the pelvis and infiltrating the bladder or the rectal mucosa (stage IVa), or with extrapelvic peritoneal metastases (stage IVb) and distant metastases (stage IVc according to the new International Federation of Gynaecology and Obstetrics (FIGO) 2023 endometrial cancer classification [3], previously classified as stage IVb).

Although the role of surgery in early-stage endometrial cancer is well established [4], its role in stage IV disease remains controversial. This is particularly relevant for patients with peritoneal metastasis who may present unresectable disease according to the surgical criteria used for advanced ovarian cancer with peritoneal spread [5], especially in the presence of extra-abdominal metastases. In this context, the hypothesis of applying the surgical algorithm [6,7] of the advanced ovarian cancer to stage IV endometrial cancer with peritoneal spread, opting for neoadjuvant chemotherapy and interval debulking surgery, appears to be a reasonable choice over primary debulking surgery. This option has been investigated in several retrospective studies [8,9,10,11]. According to the European Society of Gynecological Oncology (ESGO) guidelines [4], maximal cytoreduction should only be considered if a complete macroscopic resection can be achieved with acceptable morbidity. If upfront surgery is not feasible or acceptable and neoadjuvant chemotherapy is given, interval debulking surgery can only be considered if there is a response to chemotherapy. In both the adjuvant and neoadjuvant settings, the preferred regimen remains carboplatin/paclitaxel, as recommended by the National Comprehensive Cancer Network (NCCN) guidelines [12] and supported by the results of the GOG-0209 trial [13].

Nevertheless, due to the rarity of this condition, retrospective studies on advanced endometrial cancer treatment generally include stages III and IV patients in the same survival analysis. Stages IVa and IVb are usually merged, and there is a lack of precise descriptions of the initial disease extent when stage IVb is analyzed separately. Therefore, the guidelines do not provide clear guidance on the management of patients with endometrial cancer who present peritoneal carcinomatosis.

The aim of this systematic review and meta-analysis was to analyze the outcomes of primary debulking surgery and platinum-based adjuvant chemotherapy versus platinum-based neoadjuvant chemotherapy and interval debulking surgery in patients with advanced endometrial cancer and peritoneal carcinomatosis.

## 2. Methods

### 2.1. Database Queries and Study Selection

This systematic review was conducted according to the Preferred Reporting Items for Systematic Reviews and Meta-Analyses (PRISMA) guidelines [14] and aimed to answer the following research question: “*What is the impact of primary vs. interval debulking surgery on the prognosis of patients diagnosed with advanced endometrial cancer with carcinosis?*”. The study was registered in PROSPERO on 3 July 2023 (code: CRD42023438602).

The literature review was conducted by systematically searching the library databases PubMed, Scopus, WOS, and the Cochrane Library. The last updated search was conducted on 30 June 2024, using a combination of relevant key terms such as “advanced endometrial cancer”, “uterine tumor”, “carcinosis”, “neoadjuvant chemotherapy”, “platinum”, “debulking surgery”, and “cytoreductive surgery”. Details of the construction and full search are reported in the Supplementary Materials.

Inclusion criteria were as follows: (i) possibility to extract survival data (progression-free survival, overall survival) of patients with stage IVb endometrial cancer (according to FIGO 1988 or 2009) [15,16] with peritoneal carcinomatosis, regardless of tumor histotype, with or without extra-abdominal metastases; (ii) studies including patients treated with primary debulking surgery followed by platinum-based adjuvant chemotherapy (at least 90% of cases) with or without associated adjuvant radiotherapy, or (iii) studies including patients treated with platinum-based neoadjuvant chemotherapy (at least 90% of cases) who later received interval debulking surgery followed by any adjuvant treatment; (iv) randomized controlled trials, observational prospective and retrospective studies; (v) English language.

Exclusion criteria were as follows: (i) studies that merged stages III and IV or stage IVa (patients with tumor confined to the pelvis, infiltrating the bladder and/or rectal mucosa) and stage IVb (patients with distant metastases including peritoneal carcinosis) in the same survival analyses; (ii) studies evaluating hyperthermic intraperitoneal chemotherapy (HIPEC) in endometrial cancer; (iii) no information available on the neoadjuvant or adjuvant chemotherapy regimen; (iv) neoadjuvant radiotherapy, no adjuvant treatment or only adjuvant radiotherapy or hormonal therapy after surgery in primary cytoreduction; (v) congress abstracts, case reports, editorials, letters to the editor, reviews, meta-analyses without any new patient data and book chapters.

### 2.2. Data Extraction, Quality Assessment, and Statistical Analysis

The quality of the selected studies was assessed using the Newcastle–Ottawa Scale instrument [17]. Data were extracted, summarized, and reported in the tables. Statistical analyses were conducted in R version 4.4.0 (2024-04-24 ucrt)—“Puppy Cup” [18]. Individual patient data on survival outcomes were extracted from each paper either from the tables provided in the results or Supplementary Data or extrapolated from the Kaplan–Meier curves using ScanIt Software by AmsterCHEM, (https://www.amsterchem.com/scanit.html, last accessed: 1 May 2024) and reconstructed using the KMtoIPD R package [19]. All results were stored in a text file, which was used to reconstruct the final curves and calculate the *p*-value using the log-rank test with the survminer package [20]. The significance threshold was set at a *p*-value < 0.05. Further details on study selection and data extraction are provided in the Supplementary Materials.

## 3. Results

### 3.1. Article Screening Selection, Quality Assessment, and Description of Included Studies

**Figure 1:**
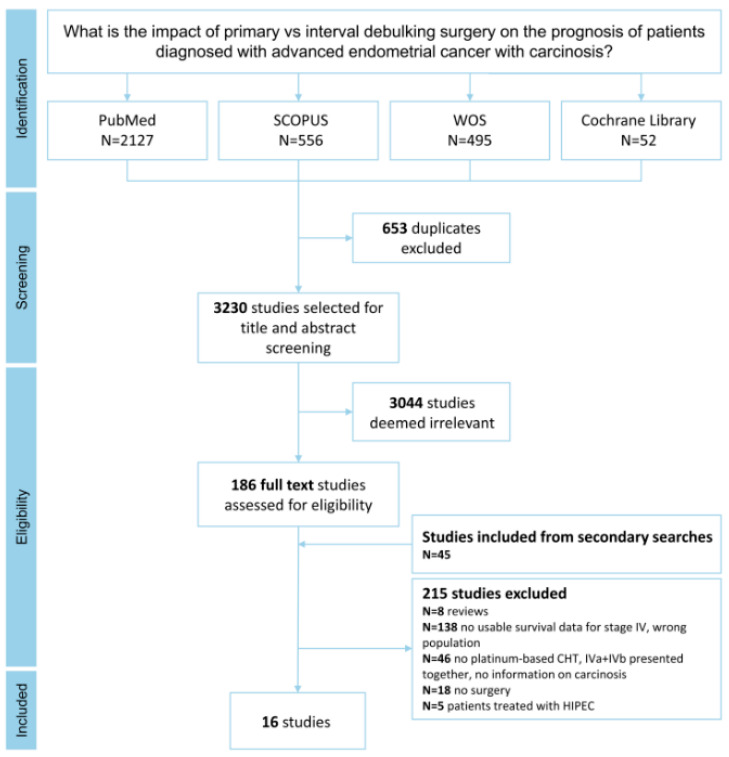
PRISMA flowchart illustrating the selection and inclusion of articles in the meta-analysis.

The results of the literature research are shown in the PRISMA flowchart (Figure 1). After removing duplicates, 3230 studies were selected for title and abstract screening, with 3044 being removed due to irrelevance toward the topic under investigation. All references were analyzed to evaluate additional eligible studies. A total of 16 studies met the selection criteria and were analyzed (Table 1, Table 2 and Table 3).

The risk of bias analysis of the included studies is reported in Supplementary Table S1. All 16 studies were rated as “good”, with 11 (68.75%) scoring 11/14 points and 5 (31.25%) scoring 12/14 points.

Most of the included studies were conducted in the United States of America (*n* = 7; 44%), followed by Asia (*n* = 5; 31%), Europe (*n* = 3; 19%), and Australia (*n* = 1; 6%). All studies except one [21], were retrospective, and the majority were single-center studies (*n* = 11; 69%). The enrollment period was 40 years (from 1980 to 2022), with publication dates ranging between 1994 and 2022, with an increase from 2010 (44%).

**Table 1:** Summary of selected studies, including only PDS patients. Legend: *n*: number; pts: patients; PDS: primary debulking surgery; RT: residual tumor; RTx: radiotherapy (may comprehend external pelvic radiotherapy, brachytherapy, whole abdominal irradiation); CT: chemotherapy; PFS: progression-free survival; OS: overall survival; MVA: multivariate analysis; HR: hazard ratio; HT: hormonal therapy; DFS: disease-free survival; N.A.: not available.

Author, Year, Country	Study Design, Enrollment Period	Age (Years)	Stages Included (Whole Cohort)	N. of Stage IVb pts with Peritoneal Dissemination	Extra-Abdominal Metastases	Abdominal Tumor Load	Histology
Bristow [22] 2000 USA	Retrospective Multicenter 1990–1998	median 65	IVb *n* = 65	65	9/65 Included in meta-analysis: 0/8	pelvis 75.4% abdominal peritoneum 49.2% omentum 47.7% bowel serosa or mesentery 36.9% upper abdomen 24.6% (whole cohort) Included in meta-analysis: pelvis 7/8 abdominal peritoneum 5/8 omentum 3/8 bowel 2/8	21 serous 22 endometrioid 22 others (whole cohort)
Landrum [23] 2009 USA	Retrospective 1990–2006	median 63	IVb *n* = 55	55	0	N.A.	29 serous 24 endometrioid 2 clear cell (whole cohort)
Lee [24] 2014 USA	Retrospective Multicenter 1980–2011	median 70	IVb *n* = 48	48	0	excluded unresectable disease 23 omentum only 25 extensive abdominal involvement	serous
Ueda [25] 2010 Japan	Retrospective 1991–2008	median 63	IVb *n* = 33	15	0	abdominal peritoneum 42% omentum 39% retroperitoneal nodes 55% bowel/mesentery 21% (whole cohort)	9 serous/clear cell 24 endometrioid (whole cohort)
Gehrig [26] 2004 USA	Retrospective 1990–2000	median 68	III–IV *n* = 24	11	0	omental involvement	serous
Watari [27] 2005 Japan	Retrospective 1982–2002	median 58	IIIC–IV *n* = 55	11	0	N.A.	12 serous/clear cell 43 endometrioid (whole cohort)
Gitsch [28] 1994 Australia	Retrospective 1988–1993	mean 73	I–IV *n* = 18	4	0	Included in meta-analysis: diaphragm 2/3 omentum 1/3	serous
Nguyen [29] 2001 USA	Retrospective 1989–1998	mean 65	I–IV *n* = 22	1	0	omental involvement	serous
Low [30] 2005 Singapore	Retrospective 1994–2003	median 62	I–IV *n* = 26	1	0	N.A.	serous
Kelly [31] 2004 USA	Retrospective 1987–2002	mean 68	I–IV *n* = 51	9	0	omental involvement	serous
**Author, Year, Country**	**Abdominal RT** **After Surgery**		**Adjuvant** **Therapy**	**AC Regimens**	**Results**		**Included Patients For Meta-Analysis ^a^**
Bristow [22] 2000 USA	26 RT = 0 cm 10 RT ≤ 1 cm 29 RT > 1 cm (4 no hysterectomy) Included in meta-analysis: 6 RT = 0 cm 1 RT ≤ 1 cm 1 RT > 1 cm		27 CT 14 CT + RTx 11 RTx 3 HT 3 no therapy 7 unknown Included in meta-analysis: 3 CT 5 CT + RTx	platinum-based	Pts who received a treatment sequence of CT followed by RTx had a median survival rate of 40.0 months, compared to only 14.0 months for pts not receiving this combination (*p* = 0.004) The median survival of all pts undergoing optimal cytoreduction (≤1 cm RT) was 34.3 months, compared to 11.0 months for patients left with suboptimal RT (*p* = 0.0001) On MVA, only age and RT ≤ 1 cm retained significance as predictors of survival		8 OS
Landrum [23] 2009 USA	48 RT ≤ 1 cm (whole cohort)		33 CT 14 CT + RTx 8 RTx	platinum-based	Median PFS for all patients (optimal and suboptimal cytoreduction) was 13 months Optimal cytoreduction was associated with a survival advantage with an HR of 2.4 At 2 years, OS for all patients treated with PDS and adjuvant CT was 53%		47 OS
Lee [24] 2014 USA	22 RT = 0 cm 14 RT < 1 cm 4 RT = 2–5 cm 5 RT > 5 cm 3 RT unknown (whole cohort)		19 CT 16 CT + RTx 5 RTx 8 no therapy	platinum-based	At 5 years, DFS and OS rates were 12% and 19% On MVA, among pts treated with CT, optimal surgical cytoreduction (HR 0.09, 95% CI 0.02–0.35) and RTx (HR 0.36, 95% CI 0.15–0.80) were associated with a decreased rate of recurrence or progression Optimal cytoreductive surgery (HR 0.09, 95% CI 0.02–0.38) was the only significant prognostic factor for OS when the model was adjusted for age		35 PFS
Ueda [25] 2010 Japan	10 RT ≤ 2 cm 5 RT > 2 cm		15 CT	platinum-based at least 90% ^c^	Median OS RT ≤ 2 cm was 19 months vs. 6 months if RT > 2 cm (*p* = 0.0007) Median PFS RT ≤ 2 cm was 10 months vs. 1 months if RT > 2 cm (*p* = 0.0003)		15 OS & PFS
Gehrig [26] 2004 USA	7 RT = 0 cm 4 RT > 0 cm Included in meta-analysis: 6 RT = 0 cm, 4 RT > 0 cm		10 CT 1 RTx	platinum-based	In the whole cohort (stages III and IV) time to progression for pts receiving RTx was 5.3 months as compared with 12.4 months for pts receiving CT (*p* = 0.01) Mean time to death for the RTx group was 8 months compared to 18 months in the CT group (*p* = 0.04)		10 OS
Watari [27] 2005 Japan	N.A.		11 CT	platinum-based	5-year survival rate of stage IV pts was 20%		11 OS
Gitsch [28] 1994 Australia	2 RT < 2 cm 2 RT > 2 cm Included in meta-analysis: 2 RT < 2 cm, 1 RT > 2 cm		1 CT 2 CT + RTx 1 no therapy	platinum-based	Of the pts with stages III and IV disease, 4 of 12 are alive with no evidence of disease after a mean follow-up of 22.5 months (range, 8–45 months) Eight of 12 women who received CT are alive with no evidence of disease, 4 of whom had stage III or IV disease		3 OS
Nguyen [29] 2001 USA	RT = 0		1 CT	platinum-based	The projected 2-year survival was 40% for pts with stages III and IV as compared with 60% for pts with stages I and II		1 OS
Low [30] 2005 Singapore	minimal RT		1 CT + RTx	platinum-based	The OS at 5 years was 72.9% for stage I, 100% for stage II, 58.9% for stage III, and 0% for stage IV		1 OS
Kelly [31] 2004 USA	8 RT < 2 cm 1 RT > 2 cm		at least 8 CT ^b^	platinum-based	Eight of the 10 stage IIIC/IV pts either progressed or recurred, and their median DFS was 6 months (range, 0–24 months)		9 OS

^a^ pts meeting all inclusion criteria with available individual survival data; ^b^ one of the pts included may have not received adjuvant CT (not clear in the text); ^c^ a minority of patients may have received adjuvant irinotecan alone or oral medroxyprogesterone acetate (not clear in the text if these few cases belong to the group of pts included in our meta-analysis).

**Table 2:** Summary of selected studies including only IDS patients. Legend: *n*: number; pts: patients; IDS: interval debulking surgery; RT: residual tumor; PR: partial response; PD: progressive disease; SD: stable disease; CT: chemotherapy; PFS: progression-free survival; OS: overall survival; MVA: multivariate analysis; UVA: univariate analysis; HR: hazard ratio; NACT: neoadjuvant chemotherapy; N.A.: not available.

Author, Year, Country	Study Design, Enrollment Period	Age (Years)	Stages Included (Whole Cohort)	N. of pts Stage IVb with Peritoneal Dissemination	Extra-Abdominal Metastases	Abdominal Tumor Load	Histology	N. of NACT Cycles	Response to NACT
Vandenput [21] 2009 Belgium	Prospective 1999–2007	median 65	IVb *n* = 30	30	some may have had pleural effusion	N.A.	27 serous 2 endometrioid 1 clear cell (whole cohort)	3–4	2 CR 20 PR 6 SD 2 PD (no IDS)
Lim [32] 2022 Korea	Retrospective Multicenter 2008–2020	median 56	IIIC–IVb *n* = 32	10	8/10	“unresectable”	2 serous 5 endometrioid 3 carcinosarcoma	median 6	10 PR
Jani [33] 2021 USA	Retrospective 2003–2019	median 63	III–IV *n* = 40	22 ^b^	pleural effusion 9/40 lung metastasis 3/40 liver 4/40	carcinomatosis 32.5% omental caking 32.5% ascites 55% extensive nodal involvement 55% bowel/mesentery 7.5% (whole cohort) Included in meta-analysis: at least omentum 22/22	18 serous 2 endometrioid 6 clear cell 9 carcinosarcoma 2 mixed 3 undifferentiated (whole cohort)	25 pts 3–4 15 pts ≥ 5 (whole cohort)	3 CR 29 PR 6 SD 2 PD (whole cohort)
**Author, Year, Country**	**NACT Regimens**	**Abdominal RT After Surgery**		**Adjuvant Therapy**	**AC Regimens**	**Results**		**Included Patients for Meta-Analysis** **^a^**	
Vandenput [21] 2009 Belgium	platinum-based	22 RT = 0 cm 2 RT < 1 cm		24 CT	22 platinum-based 2 “switch” to other type	Histopathological features of chemoresponse in both uterus and omentum were related to a better PFS (*p* = 0.017, HR = 0.785) and OS (*p* = 0.014, HR = 0.707) The use of NACT resulted in a high rate (80%) of optimal IDS		24 OS & PFS ^c^	
Lim [32] 2022 Korea	platinum-based	9 RT = 0 cm 1 RT ≤ 1 cm		10 CT	9 platinum-based 1 ifosfamide–paclitaxel	On MVA, non-endometrioid histology and RT after IDS were independent poor prognostic factors for PFS (adjusted HR 7.322, *p* < 0.001; and 5.934, *p* = 0.001, respectively) On UVA non-endometrioid histology was the only factor associated with worse OS (adjusted HR 4.523, *p* = 0.0032)		10 OS & PFS	
Jani [33] 2021 USA	platinum-based	23 RT = 0 6 RT < 1 cm 11 RT ≥ 1 cm (whole cohort)		N.A.	N.A.	Pts with higher chemotherapy response scores had longer PFS and OS and a higher rate of complete cytoreduction		22 OS & PFS	

^a^ pts meeting all inclusion criteria with available individual survival data; ^b^ omental chemotherapy response score feasible, thus surely with peritoneal dissemination; ^c^ of the 30 total stage IVb pts, 2 showed PD after NACT and, thus, did not undergo IDS, while 4 were deemed inoperable during surgery, so they were excluded from subsequent survival analysis.

**Table 3:** Summary of selected studies, including both PDS and IDS patients. Legend: *n*: number; pts: patients; PDS: primary debulking surgery; IDS: interval debulking surgery; RT: residual tumor; RTx: radiotherapy (may comprehend external pelvic radiotherapy, brachytherapy, whole abdominal irradiation); CT: chemotherapy; PFS: progression-free survival; OS: overall survival; MVA: multivariate analysis; UVA: univariate analysis; HR: hazard ratio; NACT: neoadjuvant chemotherapy; DFS: disease-free survival; N.A.: not available.

Author, Year, Country	Study Design, Enrollment Period	Age (Years)	Stages Included (Whole Cohort)	N. of pts Stage IVb with Peritoneal Dissemination	N. of pts PDS/IDS	Extra-Abdominal Metastases	Abdominal Tumor Load	Histology	N. of NACT Cycles	Response to NACT
Bogani [9] 2019 Italy	Retrospective propensity-matched 2005–2016	PDS mean 65, IDS mean 63	IVb *n* = 30	30	15/15	0	unresectable disease in patients undergoing NACT	all serous	3–6	N.A.
Unsal [34] 2022 Turkey	Retrospective multicenter N.A.	median 64	IVb *n* = 42	42	32/10	0	omental involvement 88.1% no other information	all serous	3–8	N.A.
Rajkumar [35] 2019 UK	Retrospective multicenter 2010–2016	22 pts < 65, 23 pts ≥ 65	IIIC–IVb *n* = 45	13	6/7	1/7 IDS	PDS: omentum 2/6, pelvis 5/6, bowel 1/6, retroperitoneal nodes 4/6 IDS: omentum 5/7, pelvis 5/7, bowel 1/7, retroperitoneal nodes 1/7, upper abdomen 1/7	PDS: 3 serous, 3 endometrioid IDS: 2 serous, 3 endometrioid, 1 clear cell, 1 mixed	3–6	N.A.
**Author, Year, Country**	**NACT regimens**	**Abdominal RT after Surgery**		**Adjuvant therapy**	**AC regimens**		**Results**		**Included Patients for Meta-Analysis** **^a^**	
									**PDS**	**IDS**
Bogani [9] 2019 Italy	All platinum-based	PDS: 13 RT = 0 cm, 2 RT < 1 cm IDS: 14 RT = 0 cm, 1 RT < 1 cm		PDS: 15 CT IDS: 14 CT, 1 CT + RTx	All platinum-based except 1 treated with gemcitabine in the IDS group		Similar cytoreduction rate Median DFS was 12.0 vs. 15.3 months in the IDS vs. PDS group (*p* = 0.663) Median OS was 16.7 vs. 18.0 months in the IDS vs. PDS group (*p* = 0.349)		15 OS & PFS ^b^	15 OS & PFS
Unsal [34] 2022 Turkey	All platinum-based	PDS: 26 RT = 0, 6 RT > 0 IDS: 8 RT = 0, 2 RT > 0		PDS: 32 CT IDS: not clear	all platinum-based		Receiving NACT did not affect DFS and DSS in UVA		32 OS & PFS	10 OS & PFS
Rajkumar [35] 2019 UK	>90% Platinum-based 1 capecitabine in the whole NACT cohort	PDS: 5 RT ≤ 1 cm, 1 RT > 1 cm IDS: 6 RT ≤ 1 cm, 1 RT > 1 cm		PDS: 6 CT ± RTx ^c,d^ IDS: 7 CT ± RTx ^d^	all platinum-based		Only poor performance status (*p* = 0.035), presence of bowel disease (*p* = 0.05) and suboptimal cytoreduction (*p* = 0.006) retained significance as predictors of poor survival on MVA Suboptimal cytoreduction surgery, compared to optimal cytoreduction, showed a 3.55-fold increased risk of death independent of performance status and anatomic region with disease (HR 3.55 (95% CI 1.44–8.73), *p* = 0.006)		6 OS	7 OS

^a^ pts meeting all inclusion criteria with available individual survival data; ^b^ individual survival and RT data on 4 more pts in the PDS group were obtained by direct request to the authors; ^c^ in the PDS group (whole cohort) 27 out of 28 women received postoperative CT, so it could be (not clear in the text) that 1 out of 6 stage IVb pts treated with PDS included in our meta-analysis did not receive postoperative CT; ^d^ 30 pts in the whole cohort also received extended beam pelvic RTx (not clear which pts).

### 3.2. Clinical and Pathological Features

A total of 285 patients with stage IVb endometrial cancer and peritoneal carcinomatosis were analyzed. Of these, 197 (69%) patients underwent primary debulking surgery, and 88 (31%) underwent neoadjuvant chemotherapy and interval debulking surgery. Specifically, patients were from 12 (75%) studies [9,21,22,23,24,25,26,27,32,33,34,35] that included stages III and/or IV, and 4 (25%) studies [28,29,30,31] that included all stages. In 12 (75%) studies [9,22,23,24,25,26,27,28,29,30,31,34], all patients had stage IVb endometrial cancer without extra-abdominal metastases. Considering all studies, in 230 (81%) patients, the disease was limited to the abdomen.

Considering the available information on histotype, 142 (50%) patients had serous stage IVb endometrial cancer, specifically 113 (57.4%) in the primary debulking surgery group and 29 (33%) in the interval debulking surgery group. Eight studies [9,24,26,28,29,30,31,34] included only serous endometrial cancer, and the remaining studies included all histotypes (Table 4).

Only Vandenput et al. [21] clearly defined the extent of disease using laparoscopy before offering neoadjuvant chemotherapy with interval debulking surgery, as opposed to primary debulking surgery. In contrast, seven other studies [9,22,23,25,26,31,35] relied on imaging (computed tomography scan) and/or exploratory laparotomy. Furthermore, in eight studies [23,27,28,29,30,32,33,34], there was a lack of clarity on how patients were assessed for cytoreducibility and, thus, addressed to a specific treatment.

### 3.3. Neoadjuvant/Adjuvant Treatment and Residual Tumor

Six studies included patients submitted to neoadjuvant chemotherapy [9,21,32,33,34,35], out of which, all patients but one received at least three cycles of platinum-based chemotherapy, and 56 (64%) received adjuvant chemotherapy. The adjuvant chemotherapy was platinum-based in all but four patients. Specifically, one patient received ifosfamide–paclitaxel, another received gemcitabine, and in two patients, the regimen was not specified. In the study by Bogani et al. [9], one patient also received adjuvant radiotherapy, while in the study by Rajkumar et al. [35], it is not clear whether multimodal adjuvant treatment was given. In two of the studies reporting on neoadjuvant chemotherapy [33,34], information on adjuvant treatment could not be obtained at all.

In 11 studies addressing primary debulking surgery [9,22,23,24,25,26,27,28,29,30,34], all patients except two definitively received adjuvant chemotherapy (n = 195). Postoperative chemotherapy was platinum-based in all patients in all studies except one [25], accounting for 180 (92%) out of 195 patients. Additionally, 38 (19%) out of 197 patients also received adjuvant radiotherapy. In 11 (5.6%) patients who underwent primary debulking surgery, it is not clear whether adjuvant radiotherapy was administered (Table 4).

Upon analyzing the data, we found that postoperative residual tumor was reported in 12 studies [9,21,22,25,26,28,29,30,31,32,34,35], comprising 134 patients, of whom 110 (82%) had no macroscopic residual tumor.

### 3.4. Individual Patient Data Meta-Analysis of Survival Outcomes

**Figure 2:**
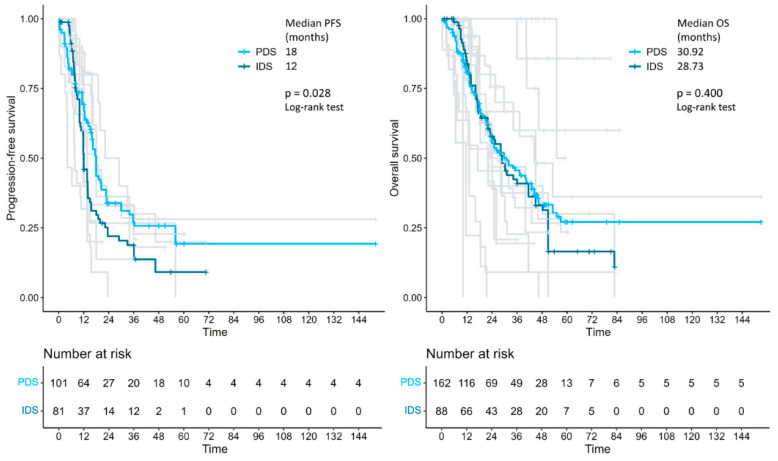
Kaplan–Meier curves, reconstructed using individual patient data extracted from the articles, showing the PFS and OS of patients with advanced endometrial cancer who received either primary debulking surgery or interval debulking surgery. PFS: progression-free survival; OS: overall survival; PDS: primary debulking surgery; IDS: interval debulking surgery.

**Figure 3:**
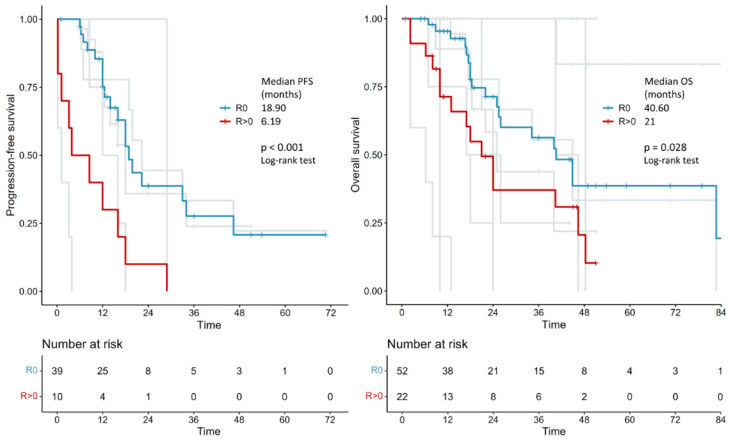
Kaplan–Meier curves reconstructed using individual patient data extracted from the articles showing the PFS and OS of patients with advanced endometrial cancer who had no macroscopic tumoral residue (R0) after surgery vs. those with residue (R > 0). PFS: progression-free survival; OS: overall survival.

In the survival analysis on 285 patients, the median progression-free survival was 18.0 vs. 12.0 months in the primary debulking surgery vs. interval debulking surgery group (log-rank *p* = 0.028). Median overall survival was 30.92 vs. 28.73 months in the primary debulking surgery vs. interval debulking surgery group (log-rank *p* = 0.400) (Figure 2). Separate survival analysis by residual tumor type was only possible in 52 (no residual tumor) versus 22 patients (residual tumor > 0) when primary debulking surgery and interval debulking surgery were considered together. Median progression-free survival was 18.9 vs. 6.19 months in the residual tumor = 0 vs. residual tumor > 0 groups (log-rank *p* < 0.001) and median overall survival was 40.6 vs. 21 months in the residual tumor = 0 vs. residual tumor > 0 groups (log-rank *p* = 0.028) (Figure 3). Unfortunately, the number of patients with individual survival data and available information regarding the residual tumor at the end of surgery was too small to perform a separate evaluation between the primary debulking surgery and interval debulking surgery groups (Supplementary Figure S1).

## 4. Discussion

### 4.1. Summary of Main Results

To the best of our knowledge, this is the first meta-analysis comparing survival outcomes of patients with stage IVb endometrial cancer with peritoneal spread treated with either primary debulking surgery and platinum-based adjuvant chemotherapy or with platinum-based neoadjuvant chemotherapy followed by interval debulking surgery. The results showed a 6-month survival benefit in terms of progression-free survival (but not in terms of overall survival) in the primary debulking surgery group compared to the interval debulking surgery group (18.0 vs. 12.0 months, *p* = 0.028). Importantly, regardless of the timing of surgery (primary or interval), complete cytoreduction (no residual tumor) results in a significant improvement in both progression-free survival (18.9 vs. 6.19 months, *p* < 0.001) and overall survival (40.6 vs. 21 months, *p* = 0.028).

### 4.2. Results in the Context of Published Literature

This systematic review highlights the lack of prospective randomized trials comparing primary debulking surgery versus neoadjuvant chemotherapy followed by interval debulking surgery in stage IVb endometrial cancer with peritoneal spread. Furthermore, the lack of clear guidance on when to choose one strategy over the other, as in the case of ovarian cancer with peritoneal metastases, represents a significant gap in the management of these patients [7]. One major issue is the absence of standardized surgical criteria for determining “optimal debulking” and the undefined thresholds for what constitutes “unresectable” or “inoperable” disease, which often leads to a switch to chemotherapy. Another critical question involves how the evaluation of “resectability” is conducted, as only Vandenput et al. [21] clearly evaluated the extent of disease with laparoscopy before offering neoadjuvant chemotherapy with interval debulking surgery. Another significant concern is the heterogeneity of adjuvant treatments that patients receive after surgery, which could potentially influence survival outcomes. Currently, there is insufficient high-quality evidence to determine whether multimodal treatment offers an oncologic benefit over chemotherapy alone in women with stage IV disease. Additionally, different characteristics of local tumor spread, such as vaginal, nodal, or parametrial involvement, may have influenced the choice of multimodal adjuvant treatments. Given these potential biases, these results should be interpreted with caution. Some authors [36] have reported a short-term overall survival advantage for patients treated with neoadjuvant chemotherapy and interval debulking surgery, attributing this benefit to fewer postoperative complications, which allow for more rapid reinitiation of medical therapies. In contrast, women treated with primary debulking surgery were at higher risk of early death but demonstrated a more favorable long-term prognosis [36]. This short-term survival advantage was confirmed in a recent systematic review by Huang et al. [10], which analyzed 5844 patients with stages III or IV endometrial cancer, including 1317 who underwent neoadjuvant chemotherapy. In our meta-analysis, only two studies [9,34] directly compared survival between the two treatment groups and found no significant advantage for either strategy. However, these studies were limited by small sample sizes and focused exclusively on serous histology. When reviewing the broader literature on this topic, we identified two additional studies [37,38] that were excluded from our analysis due to the lack of matching criteria, both of which reported contradictory results. Our results are more aligned with those of Eto et al. [37], reporting that the overall survival of patients treated with neoadjuvant chemotherapy followed by interval debulking surgery (n = 59) was comparable to that of patients undergoing primary debulking surgery (n = 279), although progression-free survival data were not reported. In contrast, the study by Wilkinson-Ryan et al. [38], which involved 44 patients, found no significant difference in median progression-free survival (primary debulking surgery: 10.4 vs. interval debulking surgery: 12 months, *p* = 0.29) or overall survival (primary debulking surgery: 17.3 vs. interval debulking surgery: 20.7 months, *p* = 0.23).

Moreover, we must emphasize the complexity of this issue and underscore the need for further research to determine the optimal treatment strategy. It is important to note that the patients included in our meta-analysis were highly selected, namely patients with advanced endometrial cancer with carcinomatosis, contrasting previous reports that included both stage IV and stage III patients.

Similarly to advanced ovarian cancer [6], and in line with our findings considering all patients, the presence of the residual tumor following surgery in endometrial cancer with peritoneal implants is one of the main prognostic factors [39,40,41]. In 2021, a systematic review and meta-analysis by Albright et al. on primary debulking surgery in the treatment of advanced-stage endometrial cancer (stages III and IV) [42] showed that submaximal (any gross residual disease vs. no residual tumor) and suboptimal (residual tumor ≥ 1 cm vs. residual tumor < 1 cm) cytoreduction were associated with worse progression-free survival and overall survival. When optimal cytoreduction is not feasible, neoadjuvant chemotherapy should be offered with the aim of achieving no residual tumor at interval debulking surgery, potentially improving prognosis while maintaining a low complication rate. In support of this concept, recently, Kanno et al. [43] found that, in stage IVb endometrial cancer, achieving resection without intra-abdominal macroscopic residue, whether before or after chemotherapy, could extend survival. The authors concluded that surgery should be pursued (primary or interval debulking surgery) when it can achieve no intra-abdominal residual tumor. Unfortunately, the lack of data on the residual tumor after primary debulking surgery or interval debulking surgery in a significant percentage of patients included in our meta-analysis (62% and 32%, respectively) prevented us from conducting a separate survival analysis based on the completeness of cytoreduction in the two treatment strategies.

### 4.3. Strengths and Weaknesses

One strength of our systematic review and meta-analysis is the strict inclusion criteria, which allowed us to focus specifically on advanced endometrial cancer with well-defined disease distribution and treatment protocols, rather than broadly including heterogenous patients as previously done by others. However, the lack of prospective studies to better define the selection criteria for primary debulking surgery or interval debulking surgery as well as the non-standardized surgical criteria for the achievement of optimal debulking in advanced endometrial cancer remain important limitations. It is worth discussing that patients with more advanced/unresectable carcinomatosis (and, therefore, with a worse prognosis) may have been referred to for neoadjuvant chemotherapy and interval debulking surgery rather than primary debulking surgery. Another limitation of our study was the inability to extract data on tumor histotype and residual tumor after surgery for a significant percentage of patients in both the primary and interval debulking surgery groups. Additionally, it is important to highlight the heterogeneity of adjuvant treatments, particularly the higher rate of postoperative radiotherapy in the primary debulking surgery group, with an unclear impact on survival outcomes. Furthermore, despite the difficulties in reconstructing survival curves, meta-analyses based on individual patient data are generally considered superior for validating and synthesizing study results [44].

### 4.4. Implications for Practice and Future Research

While a randomized controlled trial would ideally determine whether primary debulking surgery improves outcomes in “resectable” abdominal carcinomatosis of endometrial origin, the rarity of this condition makes such a trial impractical. In its place, well-designed multicentric retrospective or prospective studies are necessary. Additionally, in the era of molecular-integrated risk profiles for endometrial cancer [45,46], the role of targeted therapies, based on molecular characteristics like HER2 overexpression or mismatch repair deficiency, should be considered alongside standard chemotherapy. These innovative treatments may reduce the reliance on surgery in advanced cases.

## 5. Conclusions

The results of this meta-analysis indicate that primary debulking surgery should be considered the preferred treatment approach for advanced endometrial cancer with intra-abdominal peritoneal spread, especially in carefully selected patients where complete cytoreduction is achievable. Further prospective studies are necessary to confirm these results and to establish standardized criteria for patient selection for debulking surgery to ensure the best possible oncological outcomes.

## Figures and Tables

**Table 4:** Comparison between PDS and IDS groups of patients included in the meta-analysis, considering histology, residual tumor, and adjuvant treatment. Legend: PDS: primary debulking surgery; IDS: interval debulking surgery; RT: residual tumor; CT: chemotherapy; RTx: radiotherapy (may comprehend external pelvic radiotherapy, brachytherapy, whole abdominal irradiation); N.A.: not available.

	PDS N = 197	IDS N = 88	*p*-Value
Histology—n (%):			<0.001
Serous	113 (57.4)	29 (33.0)	
Other histotypes	3 (1.5)	13 (14.8)	
N.A.	81 (41.1)	46 (52.2)	
Residual tumor—n (%):			0.009
RT = 0	55 (27.9)	55 (62.5)	
RT > 0	19 (9.7)	5 (5.7)	
N.A.	123 (62.4)	28 (31.8)	
Adjuvant treatment—n (%):			0.002
CT	148 (75.1)	48 (54.5)	
CT + RTx	38 (19.3)	1 (1.2)	
N.A.	11 (5.6) ^a^	39 (44.3) ^b^	

^a^ at least 10 of these patients received adjuvant platinum-based chemotherapy (not clear for one patient in the study by Rajkumar et al. [35]), but it is not clear whether they also received adjuvant radiotherapy; ^b^ includes 7 patients from the study by Rajkumar et al. [35] that surely received postoperative chemotherapy ± radiotherapy (not clear in the text).

## Data Availability

Data analysis is based on published data. No new data were generated in this article.

## Supplementary Materials

The following supporting information can be downloaded at: https://www.mdpi.com/article/10.3390/cancers17061026/s1, Table S1. Quality assessment of the selected studies. The assigned Quality Rating was Good, Fair, or Poor for each study. Figure S1. Kaplan-Meier curves reconstructed using individual patient data extracted from the articles showing the PFS and OS of patients with advanced EC divided by the timing of surgery and the macroscopic tumoral residue after surgery. Reference [47] is cited in the supplementary materials.
