# Supplementary material for: Primary or Interval Debulking Surgery for Advanced Endometrial Cancer with Carcinosis: A Systematic Review and Individual Patient Data Meta-Analysis of Survival Outcomes
Source: Cancers (Basel). 2025 Mar 19;17(6):1026. doi: 10.3390/cancers17061026 (PMC11940429; doi:10.3390/cancers17061026)
Supplement: Supplementary file 1 [file cancers-17-01026-s001.zip › cancers-3494617-supplementary/Supplementary_methods_adv_search.pdf]

Supplementary methods

Database search

The search strategy was designed by an experienced librarian with input from the study authors. The detailed queries for searching the databases are presented below.

Scopus

(((ALL ("neoadjuvant therapy" OR "neoadjuvant chemotherapy" OR "preoperative chemotherapy" OR "pre-operative chemotherapy" OR "neo-adjuvant chemotherapy" OR nact OR "Cytoreduction Surgical Procedures" OR debulking OR cytoreduct* OR "radical surgery" OR "residual disease" OR "residual neoplasm")) AND ((ALL ("endometrial carcinoma*" OR "endometrial cancer*" OR "endometrial neoplasm*" OR "endometrial tumour*" OR "endometrial tumor*" OR "endometrial malignanc*" OR "uterine neoplasm*" OR "uterine carcinoma*" OR "uterine malignanc*" OR "uterine cancer*" OR "uterine tumor*" OR "uterine tumour*")) AND (ALL (("neoplasm* staging" OR "advanced stage*" OR "stage iii" OR "stage iv" OR metastatic OR carcinosis OR carcinomatosis)))))) AND (INDEXTERMS ("clinical trials" OR "clinical trials as a topic" OR "randomized controlled trial" OR "Randomized Controlled Trials as Topic" OR "controlled clinical trial" OR "Controlled Clinical Trials" OR "random allocation" OR "Double-Blind Method" OR "Single-Blind Method" OR "Cross-Over Studies" OR "Placebos" OR "multicenter study" OR "double blind procedure" OR "single blind procedure" OR "crossover procedure" OR "clinical trial" OR "controlled study" OR "randomization" OR "placebo")) OR (TITLE-ABS-KEY (("clinical trials" OR "clinical trials as a topic" OR "randomized controlled trial" OR "Randomized Controlled Trials as Topic" OR "controlled clinical trial" OR "Controlled Clinical Trials as Topic" OR "random allocation" OR "randomly allocated" OR "allocated randomly" OR "Double-Blind Method" OR "Single-Blind Method" OR "Cross-Over Studies" OR "Placebos" OR "cross-over trial" OR "single blind" OR "double blind" OR "factorial design" OR "factorial trial"))) OR (TITLE-ABS (clinical trial* OR trial* OR rct* OR random* OR blind*)) AND NOT TITLE-ABS-KEY("systematic review" OR "meta-analysis" OR review)

PubMed/Medline

("endometrial neoplasms"[MeSH Terms] OR "Uterine Neoplasms"[MeSH Terms] OR "endometrial carcinoma*"[Title/Abstract] OR "endometrial cancer*"[Title/Abstract] OR "endometrial neoplasm*"[Title/Abstract] OR "endometrial tumour*"[Title/Abstract] OR "endometrial tumor*"[Title/Abstract] OR "endometrial malignanc*"[Title/Abstract] OR "uterine neoplasm*"[Title/Abstract] OR "uterine carcinoma*"[Title/Abstract] OR "uterine malignanc*"[Title/Abstract] OR "uterine cancer*"[Title/Abstract] OR "uterine tumor*"[Title/Abstract] OR "uterine tumour*"[Title/Abstract]) AND ("antineoplastic combined chemotherapy protocols/therapeutic use"[MeSH Terms] OR "neoadjuvant therapy"[MeSH Terms] OR "neoadjuvant chemotherapy"[Title/Abstract] OR "preoperative chemotherapy"[Title/Abstract] OR "pre operative chemotherapy"[Title/Abstract] OR "neo adjuvant chemotherapy"[Title/Abstract] OR "NACT"[Title/Abstract] OR ("Cytoreduction Surgical Procedures"[MeSH Terms] OR "debulking"[Title/Abstract] OR "cytoreduct*"[Title/Abstract] OR "radical surgery"[Title/Abstract] OR "residual disease"[Title/Abstract] OR "Cytoreduction Surgical Procedures"[MeSH Terms] OR "debulking"[Title/Abstract] OR "cytoreduct*"[Title/Abstract] OR "radical surgery"[Title/Abstract] OR "residual disease"[Title/Abstract] OR "neoplasm, residual"[MeSH Terms])) AND (("randomized controlled trial"[Publication Type] OR "controlled

clinical trial"[Publication Type] OR "randomized"[Title/Abstract] OR "placebo"[Title/Abstract] OR "drug therapy"[MeSH Subheading] OR "randomly"[Title/Abstract] OR "trial"[Title/Abstract] OR "groups"[Title/Abstract]) NOT ("animals"[MeSH Terms] NOT "humans"[MeSH Terms]))

WOS

(TS=(("neoadjuvant therapy" OR "neoadjuvant chemotherapy" OR "preoperative chemotherapy" OR "pre-operative chemotherapy" OR "neo-adjuvant chemotherapy" OR nact OR "Cytoreduction Surgical Procedures" OR debulking OR cytoreduct* OR "radical surgery" OR "residual disease" OR "residual neoplasm") AND ("endometrial carcinoma*" OR "endometrial cancer*" OR "endometrial neoplasm*" OR "endometrial tumour*" OR "endometrial tumor*" OR "endometrial malignanc*" OR "uterine neoplasm*" OR "uterine carcinoma*" OR "uterine malignanc*" OR "uterine cancer*" OR "uterine tumor*" OR "uterine tumour*") AND ("neoplasm* staging" OR "advanced stage*" OR "stage iii" OR "stage iv" OR metastatic OR carcinosis OR carcinomatosis))) AND TS=((randomised OR randomized OR randomisation OR randomisation OR placebo* OR (random* AND (allocat* OR assign*)))) and Preprint Citation Index (Exclude – Database)

NOT (TI=(systematic review OR meta-analysis)) OR AB=(systematic review OR meta-analysis)

Data extraction and Quality assessment

The resulting articles were downloaded in RIS format and loaded into the Rayyan tool[1] to remove duplicates and to screen and select relevant articles. Studies were independently screened by two authors (GM, and CAC) and conflicts were resolved by discussion with a senior author (AMP). The selected articles were discussed among the authors for interdisciplinary issues. A specific request was made to the authors of the study by Bogani et al.[2] to obtain the individual patient data used in their study. All extracted data were summarized and reported in tables. Parameters of interest considered in the selected studies included: study design and enrolment period, age of patients, stage of disease, histotype, distribution of disease in the abdominal cavity, presence of extra-abdominal disease, type of treatment (primary debulking surgery or interval debulking surgery), number of cycles and regimen of neoadjuvant chemotherapy, response to neoadjuvant chemotherapy, type of adjuvant treatment, number of cycles of adjuvant chemotherapy, adjuvant chemotherapy regimen, residual tumour at the end of surgery, number of patients included for each of the two treatment strategies, survival outcomes (PFS and/or OS).

As most of the included studies were retrospective clinical studies and single-arm studies without a control group, the quality of the studies was assessed using the Newcastle–Ottawa Scale instrument [3].

Supplementary references

1. Ouzzani, M.; Hammady, H.; Fedorowicz, Z.; Elmagarmid, A. Rayyan—a Web and Mobile App for Systematic Reviews. *Systematic Reviews* **2016**, *5*, 210, doi:10.1186/s13643-016-0384-4.

2. Bogani, G.; Ditto, A.; Leone Roberti Maggiore, U.; Scaffa, C.; Mosca, L.; Chiappa, V.; Martinelli, F.; Lorusso, D.; Raspagliesi, F. Neoadjuvant Chemotherapy Followed by Interval Debulking Surgery for Unresectable Stage IVB Serous Endometrial Cancer. *Tumori* **2019**, *105*, 92–97, doi:10.1177/0300891618784785.
3. Lo, C.K.-L.; Mertz, D.; Loeb, M. Newcastle-Ottawa Scale: Comparing Reviewers' to Authors' Assessments. *BMC Medical Research Methodology* **2014**, *14*, 45, doi:10.1186/1471-2288-14-45.
