# Supplementary material for: Primary or Interval Debulking Surgery for Advanced Endometrial Cancer with Carcinosis: A Systematic Review and Individual Patient Data Meta-Analysis of Survival Outcomes
Source: Cancers (Basel). 2025 Mar 19;17(6):1026. doi: 10.3390/cancers17061026 (PMC11940429; doi:10.3390/cancers17061026)
Supplement: Supplementary file 1 [file cancers-17-01026-s001.zip › cancers-3494617-supplementary/Supplem_results.pdf]

**Supplementary Table 1.** Quality assessment of the selected studies. The assigned Quality Rating was Good, Fair, or Poor for each study.

| Article / Item | 1 | 2 | 3 | 4 | 5 | 6 | 7 | 8 | 9 | 10 | 11 | 12 | 13 | 14 | **Total score** |
|---|---|---|---|---|---|---|---|---|---|---|---|---|---|---|---|
| Bogani 2019 | ☺ | ☺ | ☺ | ☺ | ☹ | ☺ | ☺ | 😐 | ☺ | 😐 | ☺ | ☹ | ☺ | ☹ | **11** |
| Bristow 2000 | ☺ | ☺ | ☺ | ☺ | ☹ | ☺ | ☺ | 😐 | ☺ | 😐 | ☺ | ☹ | ☺ | ☺ | **12** |
| Gehrig 2004 | ☺ | ☺ | ☺ | ☺ | ☹ | ☺ | ☺ | 😐 | ☺ | 😐 | ☺ | ☹ | ☺ | ☹ | **11** |
| Gitsch 1994 | ☺ | ☺ | ☺ | ☺ | ☹ | ☺ | ☺ | 😐 | ☺ | 😐 | ☺ | ☹ | ☺ | ☹ | **11** |
| Jani 2021 | ☺ | ☺ | ☺ | ☺ | ☹ | ☺ | ☺ | 😐 | ☺ | 😐 | ☺ | ☹ | ☺ | ☹ | **11** |
| Kelly 2004 | ☺ | ☺ | ☺ | ☺ | ☹ | ☺ | ☺ | 😐 | ☺ | 😐 | ☺ | ☹ | ☺ | ☹ | **11** |
| Landrum 2009 | ☺ | ☺ | ☺ | ☺ | ☹ | ☺ | ☺ | 😐 | ☺ | 😐 | ☺ | ☹ | ☺ | ☺ | **12** |
| Lee 2014 | ☺ | ☺ | ☺ | ☹ | ☹ | ☺ | ☺ | 😐 | ☺ | 😐 | ☺ | ☹ | ☺ | ☺ | **11** |
| Lim 2022 | ☺ | ☺ | ☺ | ☺ | ☹ | ☺ | ☺ | 😐 | ☺ | 😐 | ☺ | ☹ | ☺ | ☹ | **11** |
| Low 2005 | ☺ | ☺ | ☺ | ☺ | ☹ | ☺ | ☺ | 😐 | ☺ | 😐 | ☺ | ☹ | ☺ | ☹ | **11** |
| Nguyen 2001 | ☺ | ☺ | ☺ | ☺ | ☹ | ☺ | ☺ | 😐 | ☺ | 😐 | ☺ | ☹ | ☺ | ☹ | **11** |
| Rajkumar 2019 | ☺ | ☺ | ☺ | ☺ | ☹ | ☺ | ☺ | 😐 | ☺ | 😐 | ☺ | ☹ | ☺ | ☺ | **12** |
| Ueda 2010 | ☺ | ☺ | ☺ | ☺ | ☹ | ☺ | ☺ | 😐 | ☺ | 😐 | ☺ | ☹ | ☺ | ☺ | **12** |
| Unsal 2022 | ☺ | ☺ | ☺ | ☹ | ☺ | ☺ | ☺ | 😐 | ☺ | 😐 | ☺ | ☹ | ☺ | ☹ | **11** |
| Vandeput 2009 | ☺ | ☺ | ☺ | ☺ | ☹ | ☺ | ☺ | 😐 | ☺ | 😐 | ☺ | ☹ | ☺ | ☹ | **11** |
| Watari 2005 | ☺ | ☺ | ☺ | ☺ | ☹ | ☺ | ☺ | 😐 | ☺ | 😐 | ☺ | ☹ | ☺ | ☺ | **12** |

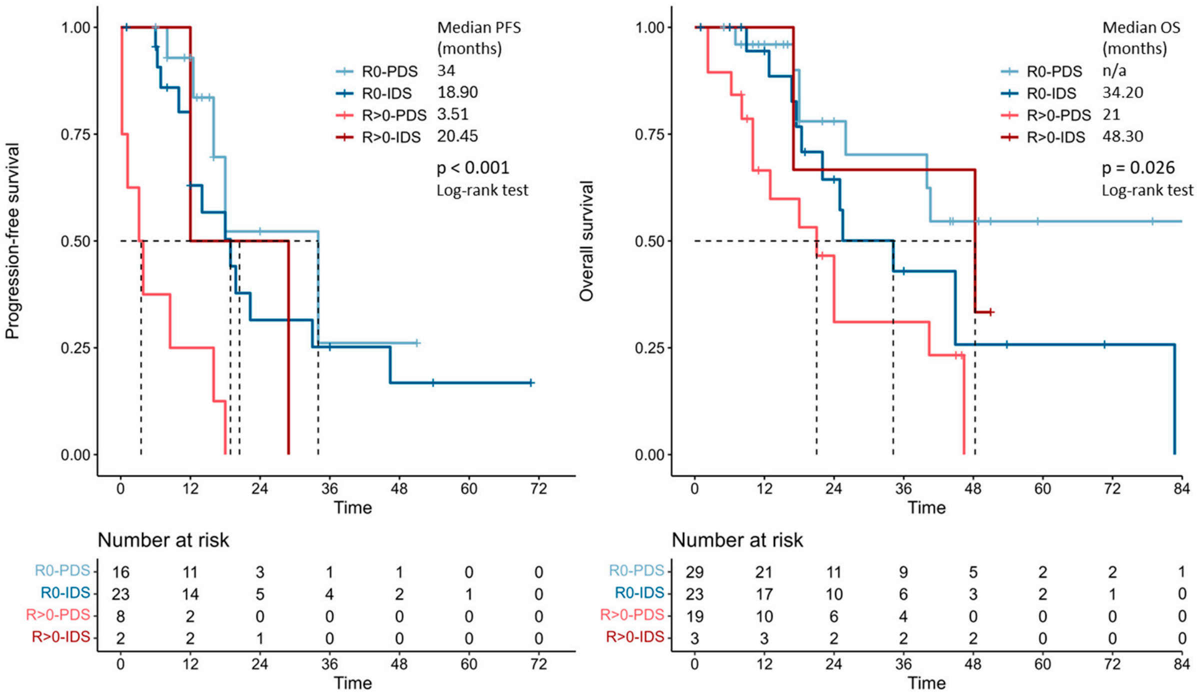


| p-value | R0-PDS | R0-IDS | R1-PDS |
|---|---|---|---|
| R0-IDS | 0.292 | | |
| R1-PDS | **<0.001** | **<0.001** | |
| R1-IDS | 0.294 | 0.745 | 0.142 |

| p-value | R0-PDS | R0-IDS | R1-PDS |
|---|---|---|---|
| R0-IDS | 0.127 | | |
| R1-PDS | **0.004** | 0.135 | |
| R1-IDS | 0.642 | 0.652 | 0.118 |

**Supplementary figure 1.** Kaplan-Meier curves reconstructed using individual patient data extracted from the articles showing the PFS and OS of patients with advanced EC divided by the timing of surgery and the macroscopic tumoral residue after surgery.
